# Do variations in Insulin-like Factor 3 (INSL3) gene affect PCOS susceptibility?

**DOI:** 10.1186/1755-8166-7-S1-P93

**Published:** 2014-01-21

**Authors:** Nuzhat Shaikh, Nalini Shah, Srabani Mukherjee

**Affiliations:** 1 National Institute for Research in Reproductive Health, J.M. Street, Parel, Mumbai- 400012, India; 2 Department of Endocrinology, Seth GS Medical College, Mumbai- 400012, India

## 

Polycystic ovary syndrome (PCOS) is a multigenic complex disorder causing metabolic and gynecologic dysfunctions in women of reproductive age. Its cardinal features include hyperandrogenemia, chronic anovulation, insulin resistance and hyperinsulinemia. Women with PCOS are at increased risk to develop long term health implications like endometrial cancer, T2DM and cardiovascular diseases. Insulin-like factor 3 (INSL3), also known as relaxin-like factor (RLF), is a member of the relaxin-like hormone family. Relaxin and INSL3 are peptide hormones with a number of important physiological roles in reproduction, regulation of extracellular matrix turnover, and cardiovascular function. INSL3 is a theca cell-secreted paracrine factor which regulates androgen production and has been implicated a role in follicle selection and resulting ovulation. In women, it is produced in lower amounts by ovarian theca and luteal cells, and circulating levels are increased in women with polycystic ovarian syndrome. This suggests that INSL3 may have a modulatory role in ovarian function. The INSL3 gene is localized on chromosome 19 and comprises of two exons and an intron. Novel and known variations in both exonic regions were investigated to understand their influence in PCOS pathophysiology. Genotyping of these polymorphisms were carried out by direct sequencing in 150 women with PCOS and 150 normal menstruating women. Clinical, biochemical and hormonal parameters were also assessed in these women. Analysis revealed association of db6523 SNP in exon 1 and rs1003887 polymorphism of exon 2 with susceptibility to PCOS. Other exonic region polymorphisms failed to show any association with PCOS pathophysiology.

